# From Plant to Skin: Exploring *Alnus glutinosa* Extracts for Cosmeceutical Applications

**DOI:** 10.3390/antiox14111275

**Published:** 2025-10-23

**Authors:** Nikolaos D. Bikiaris, Evangelia Balla, Despoina Varitimidou, Lelouda-Athanasia Koronaiou, Nikolaos Nikolaidis

**Affiliations:** 1 Laboratory of Polymer Chemistry and Technology, Department of Chemistry, Aristotle University of Thessaloniki, GR-54124 Thessaloniki, Greece; euagelia226@gmail.com (E.B.); depvar18@gmail.com (D.V.); 2 Laboratory of Environmental Pollution Control, Department of Chemistry, Aristotle University of Thessaloniki, GR-54124 Thessaloniki, Greece; koronaio@chem.auth.gr

**Keywords:** *Alnus glutinosa*, extracts, antioxidant, sun protection, cosmeceuticals

## Abstract

This study explores the photoprotective and antioxidant potential of cosmetic emulsions formulated with *Alnus glutinosa* (black alder) extracts. Extraction of bioactive compounds was performed using Soxhlet, ultrasound-assisted, and microwave-assisted techniques with ethanol and water as solvents. The phytochemical profiles of the resulting extracts were characterized via UV-Vis spectroscopy, Fourier-transform infrared spectroscopy (FTIR), and liquid chromatography–high-resolution mass spectrometry (LC-HRMS). The extracts were incorporated into oil-in-water emulsions and assessed for antioxidant activity using the DPPH radical scavenging assay, pH and viscosity stability, and color L*a*b* values. Among the extraction methods, ethanol-based Soxhlet extraction yielded the highest concentration of bioactive compounds and demonstrated superior antioxidant and photoprotective efficacy. This is the first report that evaluates the antioxidant properties of *A. glutinosa*-enriched emulsions, supporting their application as multifunctional, plant-derived cosmeceuticals for skin protection.

## 1. Introduction

Cosmeceuticals, a rapidly expanding sector within the natural personal care industry, represent a class of products that bridge the gap between cosmetics and pharmaceuticals. Natural ingredients have been utilized in dermatological applications for centuries, and their incorporation into contemporary skincare formulations is increasing. Botanical extracts possess a wide spectrum of antioxidant properties, primarily due to their rich content in phenolic compounds, flavonoids, and other secondary metabolites. Extensive screening of various plant extracts—such as *Punica granatum* (pomegranate), *Syzygium aromaticum* (clove), *Mangifera indica* (mango kernel), and *Phyllanthus emblica* (amla)—has confirmed potent ability to scavenge reactive oxygen species and free radicals using in vitro assays like DPPH and electron spin resonance (ESR). These antioxidants play a pivotal role in neutralizing oxidative stress, which is implicated in skin aging and various diseases [1,2,3]. These agents exert localized therapeutic effects, supplying essential nutrients that support skin homeostasis and contribute to the prevention of degenerative cutaneous disorders [4].

In response to growing consumer interest in plant-based, multifunctional ingredients, Alnus-derived extracts offer a sustainable and eco-conscious approach to enhancing the efficacy and commercial value of topical formulations. *Alnus glutinosa* (*A. glutinosa*), also known as black alder, is a natural antioxidant predominant in Europe, North Africa and Western Asia that can reduce oxidative stress on the skin or protect products from oxidative degradation [5,6]. The antioxidant activity of *A. glutinosa* extracts from leaves and bark is mainly attributed to its rich composition of polyphenolic compounds, including diarylheptanoids, tannins, and flavonoids. These compounds are not just free radical scavengers, but also modulators of cellular oxidative stress responses, making them efficient bioactives with therapeutic and preventative benefits against skin oxidative damage, inflammation and aging [7]. Evidence of antioxidant activity was reported by Dinić et al., who isolated two diarylheptanoids from the bark of *A. glutinosa*—platyphylloside and a coumaroyl derivative thereof. The results obtained showed that the substances drastically reduced reactive oxygen species (ROS) production both in cancer and healthy cells exposed to doxorubicin and cisplatin [8]. Similarly, Skrypnik et al., using water extracts of *A. glutinosa*, reported a total phenolic content up to 29.0 ± 5.3 mg g^−1^ and radical scavenging activity as high as 276 ± 29 mg g^−1^, outperforming pine and oak extracts [9,10].

In addition to their notable antioxidant activity, *A. glutinosa* extracts demonstrate potential for photoprotection, attributed to their rich phenolic content. Compounds such as polyphenols, flavonoids, and tannins possess structural features—namely conjugated double bonds and aromatic rings—that enable absorption of UVA and UVB radiation, indicating their capacity to protect the skin against sun and UV-induced damage. Although the sun protection efficacy of *A. glutinosa* extracts in cosmetic emulsions has not yet been systematically evaluated, their compositional similarity to other polyphenol-rich botanical extracts—commonly utilized as natural SPF enhancers—suggests considerable potential for photoprotective applications. In the study of Skrypnik et al., the levels of total flavonoids (as much as 21.5 mg g^−1^ in alder bark) and proanthocyanidins (3.8 mg g^−1^) were compared to high radical scavenging activity, a secondary marker of photoprotective capacity. This is in accordance with more comprehensive research which has established that such compounds are able to block UV-induced oxidative stress in cultured skin cells [6]. Mechanistically, antioxidants cancel out the deleterious effects of UV radiation by quenching ROS produced in exposed skin. ROS are key mediators of UV-induced skin damage and contribute to collagen degradation, inflammation, and photoaging Antioxidants that reduce ROS can therefore significantly augment photoprotection of the skin and retard photoaging [11,12]. Furthermore, Dinić et al. [8] demonstrated that the diarylheptanoids of *A. glutinosa* not only suppressed ROS but also preserved mitochondrial integrity as well as preventing apoptosis in normal cells exposed to chemotherapeutic stress. The latter cytoprotective effect can also be extrapolated to protection from UV-induced damage in cells, further confirming the photoprotective effect of these compounds [13].

This study aimed to optimize the extraction conditions for *Alnus glutinosa* and evaluate the incorporation of its extracts into cosmetic emulsions. Given their phenolic profile, further investigation of the extracts’ role in antioxidant activity and UV protection in cosmetic formulations is justified. Both conventional (cold maceration and reflux boiling) and advanced (ultrasound-assisted and microwave-assisted) extraction techniques were assessed. The phytochemical profile of the extracts was analyzed using LC–HRMS for qualitative characterization of bioactive compounds. Spectroscopic methods, including UV-Vis and FTIR, were employed for further characterization. Antioxidant activity was evaluated via the DPPH assay. To the best of our knowledge, while *A. glutinosa* has been studied in the pharmacy field [14,15] no peer-reviewed publication to date investigates the use of isolated extract in cosmeceutical formulations.

## 2. Materials and Methods

### 2.1. Chemicals and Extract Isolation Methods

Foliage of *Alnus glutinosa* was obtained commercially from a local supplier (Kilkis, Greece) and dried in the air (at 25 °C in the dark, using still air for 3 days). The materials were then pulverized into a fine powder using a mechanical grinder and stored in airtight bags for further analysis. The preparation of extracts from *A. glutinosa* was performed using three different methods, and each test was carried out in triplicate. After optimizing each extraction process, isolated extracts were analyzed by UV-Vis spectroscopy. All the processes were performed using a quantity of powdered foliage at 5 g per 100 mL of solvent, while all other reagents employed were of analytical grade.

#### 2.1.1. Ultrasound Assisted Extraction

A total of 5 g of the plant samples were taken and added to 100 mL EtOH taken in the beaker. The beaker was covered using aluminum foil to prevent loss of solvent by evaporation. The ultrasonic MS3 MicroTip3 probe (Maximum amplitude: 180, W/cm^3^: 460) of Ultrasonic Processor UP100H (Controla S.A, Thessaloniki, Greece) was positioned inside the beaker and the solution was subjected to ultrasound at a frequency of 30 kHz at 25 °C and 70 °C for a predetermined duration of 60 min. Once the extraction ended, ultrasonication was stopped, and the supernatant liquid was vacuum filtered to isolate the pure extract. The filtrate was tightly closed and stored at low temperature for future reference. The optical density was determined with the help of UV–VIS spectrophotometer.

#### 2.1.2. Extraction in Soxhlet Extractor

For the reflux boiling method using a Soxhlet extractor, 5 g of *A. glutinosa* powder was placed in a round-bottomed flask and weighed, and reflux extraction was performed at three different conditions to optimize the process. The apparatus was heated on a laboratory electric heating mantle at ambient pressure. Specifically, deionized water (DI) was employed as solvent at 70 °C and 90 °C, respectively, for 3 h, as well as pure ethanol at 70 °C for 1 h (Table 1). Once the boiling ended, the supernatant liquid was vacuum filtered to isolate the pure extract.

#### 2.1.3. Microwave Assisted Extraction

A domestic microwave oven (NN-S255W, Panasonic, Tokyo, Japan) was used in the current study with a total capacity of 1100 W. Plant samples (5 g) were mixed with the DI (100 mL) and placed in a beaker, and then, each beaker was inserted alone into the microwave oven.

The mixtures were subjected to microwave irradiation at 330 W, following the method described by Pan et al. [16] with slight modifications to reach a total irradiation time of 3 min: 45 s of power on, followed by 30 s off, and then an additional 15 s of power on. After every 60 s of irradiation, the sample was allowed to cool down to room temperature.

### 2.2. Characterization of Extracts

#### 2.2.1. Gravimetric Analysis

At the end of each extraction process, the samples were filtered, and the solvent was evaporated using rotary evaporation. Purified extracts were placed into refrigeration overnight and were further lyophilized using a Scavnac freeze-drier system (Coolsafe 110-4 Pro, LaboGene Scandinavia, Hovedstaden, Denmark) at −80 °C for 24 h. Crude extracts were stored at 4 °C. The yield of the extract was calculated by determining the weight of the dried extract per gram of plant material used, using the following equation. All measures were performed in triplicate.

(1) $$yield\;of\;extract\;\%=\frac{extract\;obtained\;(g)}{\;amount\;of\;plant\;powder\;used}\;*\;100\;\;$$

#### 2.2.2. UV-Vis Spectrometry

The quantitative evaluation of isolated extracts was executed through UV-Vis spectrometry. A Shimadzu UV-1800 spectrophotometer equipped with UV Probe ver. 2.61 software (Shimadzu, Kyoto, Japan) was used for obtaining the absorption spectra after suitably diluting the extracts. The UV–VIS spectra of the coloring compounds from each plant material were recorded in the visible range between 400 and 700 nm. The extracted solutions were then analyzed by measuring their absorbance values at either the wavelength corresponding to the maximum absorbance (λmax) or at a designated specific wavelength.

#### 2.2.3. Fourier-Transform Infrared Spectroscopy (FTIR)

FTIR spectra were obtained using a Perkin-Elmer FTIR spectrometer (Perkin Elmer, Waltham, MA, USA), model SPECTRUM 1000, using KBr tablets. The spectra of the extracts were taken in the range 4000–500 cm^−1^, with a resolution 4 cm^−1^ and 16 scans, and were baseline corrected and converted to absorbance mode using the Spectrum v.5.3.1 (2004) software.

#### 2.2.4. LC-Orbitrap-HRMS

Instrumental analysis was performed using a Q Exactive Focus Orbitrap LC-MS/MS system (Thermo Fisher Scientific, Bremen, Germany). The liquid chromatography (LC) setup included a Vanquish Flex ultra-high-performance liquid chromatography (UHPLC) system equipped with binary pumps, a temperature-controlled autosampler, and a column compartment. Separation was carried out on a Thermo Hypersil GOLD aQ column (50 × 2.1 mm, 1.9 μm) with a holder and cartridge pre-filter, maintained at a constant temperature of 40 °C throughout the analysis.

The mobile phases consisted of water (A) and methanol (B), both acidified with 0.1% (*v*/*v*) formic acid (FA). The gradient program began with 10% B, held for 1.5 min; then, it was increased to 60% B over the next 2.5 min, followed by a rise to 70% B over 4 min, reaching 100% at 11 min, which was maintained for 1 min. The mobile phase composition was then restored to the initial conditions within one minute and held for 2 min to allow re-equilibration. The total run time was 15 min at a constant flow rate of 200 µL/min. The injection volume was set to 5 µL, and the autosampler tray was maintained at 10 °C.

For mass spectrometry, ionization was achieved via a heated electrospray ionization (HESI-II) probe, Ion Max model, using polarity switching mode. Instrument settings followed the system’s default parameters optimized for an LC flow rate of 0.2 mL/min: tube lens voltage at 110 V; sheath, auxiliary, and sweep gas flow rates at 45, 10, and 2 arbitrary units (au), respectively, supplied by a high-purity nitrogen generator (Peak Scientific Genius 1022, Inchinnan, UK); spray voltages of 3.5 kV in positive mode and 2.7 kV in negative mode; and an S-lens RF level set at 50. Finally, the auxiliary gas heater temperature was 400 °C, the capillary temperature was set at 320 °C. Precursor ions (full scan, MS1) were scanned in the 100–1000 *m*/*z* range with a resolving power of 70,000 (at *m*/*z* 200). To facilitate structural elucidation, data-dependent acquisition (dd-MS2) was employed in parallel, operating in Discovery Mode to trigger MS2 events based on the intensity of precursor ions. Fragmentation of the selected ions was achieved using stepped normalized collision energies of 20, 35, and 50 eV, and MS2 spectra were recorded at a resolution of 35,000. Instrument control and initial qualitative analysis were carried out using Xcalibur software version 4.1.

##### Data Processing in Compound Discoverer

Non-target analysis was performed using the commercially available Compound Discoverer 3.3 software (Thermo Scientific, Waltham, MA, USA). Multiple processing nodes were applied to develop a dedicated screening workflow for deconvoluting raw Orbitrap MS data (Figure S1). Sample files were imported and labeled as solvent blanks, samples, or quality controls (QC). The ready-to-use workflow titled “Food Research w Stats Unknown ID w Online and Local Database Searches” was selected with minor modifications. Spectra were filtered in the “Select Spectra” node based on criteria including total intensity above 1 × 10^6^, signal-to-noise ratio greater than 10, and acquisition times between 0.5 and 14 min. Retention time (RT) alignment was performed using the adaptive curve algorithm, with a maximum allowed RT difference of 0.1 min between samples; fallback alignment was executed via a linear model. Mass features passing these parameters were introduced into the “Detect Compounds” node for ion and peak detection, followed by grouping across all samples with a retention time tolerance of 10 s and a composition match threshold of 25%. Background signals were minimized by filtering out peaks with a sample-to-blank intensity ratio below 5:1. Missing data points from misalignment were corrected automatically using the “Filling Gaps” node. For compound annotation, the “Search mzCloud,” “Search ChemSpider,” “Search Mass Lists,” and “Predict Compositions” nodes were applied. The “Search mzCloud” node compared MS and MS2 spectra against the online mzCloud database using the HighChem HighRes algorithm. The “Search ChemSpider” node searched selected libraries including ACToR (Aggregated Computational Toxicology Resource), FDA databases (Structured Product Labeling index and UNII-NLM), FooDB, and Phenol-Explorer. Elemental composition prediction was limited to a maximum of C200 H800 Br5 Cl4 F5 N5 O80 P5 Si in the “Predict Compositions” node. The mass tolerance for all searches was set to 5 ppm. Post-annotation, additional filtering retained only compounds with mzCloud matching scores greater than 70%, corresponding to a confidence identification level of 2a according to Schymanski et al. (2014) [17].

### 2.3. Emulsion Preparation

**Figure 1:**
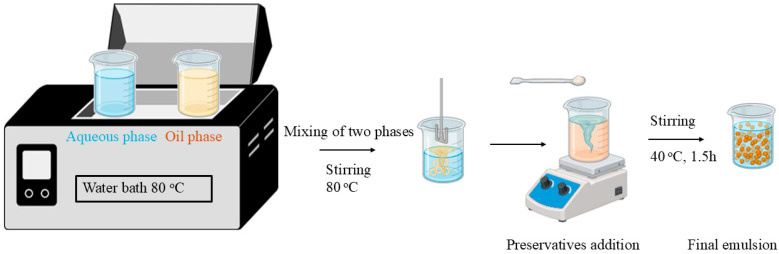
Illustration of the experimental procedure for the preparation of blank emulsions and emulsions containing *A. glutinosa* extracts.

The O/W emulsions were prepared herein following a technique from previous works [18,19] that involved three stages: (1) preparation of the aqueous phase, (2) preparation of the oily phase and (3) mixing of the two phases until the formation of a stable emulsion. The experimental procedure is illustrated in Figure 1. All ingredients (Table 2) used are listed from FDA as safe and have been approved for cosmetic applications. Each isolated extract was used for the preparation of the corresponding emulsion, while an additional cream was also prepared serving as reference sample, without extract.

(1) Oil phase (23%) was prepared in a beaker with olive oil (11%), cetylstearyl alcohol (2%), cetyl alcohol (2%), polysorbate-60 (2%), stearic acid (2%), shea butter (2%) and finally the beeswax (2%).
(2) The two beakers were placed in a water bath with the temperature set at 80 °C until all ingredients were completely homogenized. The oil phase was slowly added to the aqueous phase while stirring at 600 rpm using a 2020 RZR (Heidolph, Schwabach, Germany) mechanical stirrer, with the temperature maintained at 80 °C. Once the oil phase was fully incorporated, the heating was stopped, but stirring continued in the water bath. Stirring lasted approximately 1.5 to 2 h, until the emulsion (cream) was fully formed. Since phenoxyethanol (1%) and ethylhexylglycerin (1%) are volatile preservatives, they were added only after the temperature dropped below 40 °C. The resulting emulsions were then transferred into plastic containers and stored in a cool, dark environment.

### 2.4. Analysis of Emulsion Properties

#### 2.4.1. pH and Viscosity Stability

The stability of the prepared emulsions was evaluated by monitoring pH and viscosity over periods of 1, 7, 14, 30, 60 and 90 days following preparation. The pH was measured by immersing a microprocessor pH sensor (WTW pH 535, Gemini BV, Apeldoorn, The Netherlands) directly into the emulsions. For viscosity measurements at 50 and 100 rpm, a rotational Visco Star plus viscometer (Fungilab, SA, Barcelona, Spain) was employed. After testing various spindles, spindle R3 was selected as the most appropriate and was immersed to a consistent depth in the plastic containers during measurement [20].

#### 2.4.2. Color Measurement

The samples were analyzed at room temperature for color variations using a portable MiniScan XE Plus spectrophotometer (HUNTERLAB, Washington, VA, USA). Color evaluation was conducted by measuring changes in the Commission Internationale de l’Éclairage (CIE) LabCh components. This five-dimensional color space includes L* (lightness), ranging from 0 (black) to 100 (white); a* representing redness (positive values) or greenness (negative values); b* representing yellowness (positive values) or blueness (negative values); and C* (chroma, indicating color saturation) and H* (hue angle) [21].

#### 2.4.3. Antioxidant Study

The antioxidant capacity of the emulsions was evaluated using the DPPH (2,2-diphenyl-1-picrylhydrazyl) assay. A 50 mg/L DPPH solution in ethanol (Lot# STBH7297, Sigma Aldrich^®^, Burlington, MA, USA) was prepared, and emulsion solutions were made at 1% *w*/*v* in ethanol. Subsequently, 1 mL of each emulsion solution was mixed with 3 mL of the DPPH solution and sonicated for 30 min. To prevent light exposure during the reaction, all flasks were securely wrapped in aluminum foil. A blank control was prepared by mixing 1 mL of pure ethanol with 3 mL of DPPH solution. Absorbance measurements were conducted using a UV-Vis spectrophotometer equipped with a W-lamp, recording at the DPPH absorbance maximum of 517 nm. Measurements were recorded every 30 min over a total period of 90 min. Between measurements, all samples were kept in darkness to avoid photo-degradation of the free radical. A decrease in absorbance indicated increased free radical scavenging activity of the emulsions. The antioxidant activity was calculated using the following equation:

(2) $$Free\;radical\;scavenging\;activity\;\left(\%\right)=\frac{A_{c}-A_{S}}{A_{C}}\;\;\;\;$$

where A_S_ and A_C_ represent the absorbance of each sample and of the control sample (no cream), respectively.

### 2.5. Statistical Analysis

Extraction yields and CIELAB color space values were analyzed using Levene’s test for verification of homogeneity of variances, followed by one-way ANOVA with Tukey’s correction for multiple post hoc comparisons. Data on pH, viscosity and antioxidant activity were analyzed using repeated measures ANOVA, applying the Greenhouse–Geisser correction to adjust for violations of the sphericity assumption. Post hoc pairwise comparisons were adjusted using the Bonferroni correction, while Tukey’s correction was used for multi-group comparisons. All data are presented as mean ± SD and *p* < 0.05 was considered statistically significant. No normality tests were performed since the number of samples is three, making such tests unreliable and insufficient for assessing distributional assumptions. All analyses were performed using IBM SPSS Statistics v28.0.

## 3. Results

### 3.1. Assessment of Extract Properties

The extractive yields of *Alnus glutinosa* samples obtained through different extraction methods are presented in Table 3. Overall, a highly significant effect of the extraction method on the extractive yields was observed (*p* < 0.001). In more detail, all extraction methods differed significantly from each other (*p* < 0.001) Soxhlet extractions with ethanol (Soxhlet 3) produced the highest yields, namely 42.50 ± 1.10%, highlighting the efficacy of ethanol under prolonged hot extraction conditions. In contrast, Soxhlet extractions (Soxhlet 1 and 2) with water yielded moderate extraction efficiency. Ultrasound-assisted extractions with ethanol at 25 °C and 70 °C yielded 8.20 ± 0.35% and 14.60 ± 0.50%, respectively, indicating that higher temperatures enhance solvent penetration and solute diffusion. Microwave-assisted extraction with water produced the lowest yields (4.80 ± 0.20%), likely due to limited extraction time, lower solvent efficacy, or degradation of thermolabile compounds under the applied conditions.

**Figure 2:**
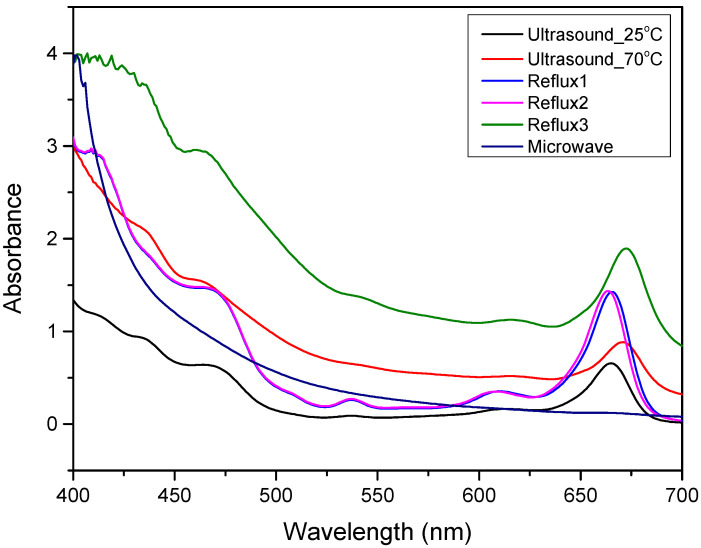
UV–VIS spectrum for *Alnus glutinosa* extracts via ultrasound at 25 and 70 °C, reflux boiling and microwave-assisted extraction.

The UV-Vis spectra of *Alnus glutinosa* extracts obtained through ultrasound, reflux boiling, and microwave-assisted methods were recorded in the wavelength range of 400–700 nm, as shown in Figure 2. This range covers the visible spectrum, which is particularly relevant for detecting chromophoric compounds. Among the evaluated methods, Soxhlet extraction yielded the highest overall absorbance values across the visible range, indicating greater extraction efficiency. Ultrasound-assisted extraction showed moderate absorbance, while microwave-assisted extraction resulted in the lowest absorbance intensity. Distinct absorption peaks near 665 nm were observed for all methods except microwave-assisted extraction.

LC–HRMS analysis was performed on two Alnus glutinosa Soxhlet extracts (Soxhlet 2 and 3) for qualitative characterization of their phytochemical composition, as presented in Table 4. Major compounds identified included quinic acid (C_7_H_12_O_6_), 3,4,5-trihydroxycyclohex-1-ene-1-carboxylic acid (C_7_H_10_O_5_), neochlorogenic acid (C_16_H_18_O_9_), and docosahexaenoic acid ethyl ester (C_24_H_36_O_2_). The extracts were also rich in natural antioxidants such as polyphenols, triterpenoids, and flavonoids. Notable compounds detected included miquelianin, gallic acid, cafestol, quercetin-3β-D-glucoside, and luteolin. Minor constituents like fatty acids and amino acids were also present, adding to the extracts’ chemical complexity. Among diarylheptanoids, only (1E)-1,7-bis(4-hydroxyphenyl)hept-1-en-3-one (C_19_H_20_O_3_) was detected. Full compound data are listed in Table S1.

**Figure 3:**
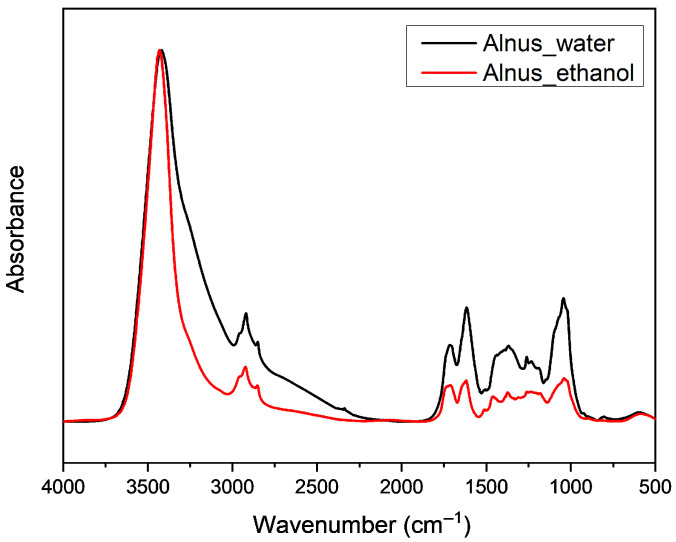
FT-IR spectrum of *Alnus glutinosa* reflux extracts in water and ethanol.

The impact of water and ethanol (EtOH) as solvents in reflux boiling was assessed using FTIR spectroscopy, with spectra presented in Figure 3. Both extracts displayed a characteristic broad band near 3430 cm^−1^, corresponding to hydroxyl (–OH) group stretching, indicative of hydrogen bonding. The ethanol extract showed a sharper and more defined –OH stretching band, while the water extract exhibited a broader, less distinct peak. A triplet signal in the 3000–2800 cm^−1^ region confirmed the presence of C–H stretching vibrations, suggesting methylene groups and minor carbonyl (C=O) contributions. Strong absorption in the 1620–1600 cm^−1^ region and a weaker band at 1260 cm^−1^ provided additional evidence of aromatic conjugated ketones. Further absorption bands at 1460, 1370, and within the 1200–900 cm^−1^ range indicated C–H bending and stretching vibrations. Overall, FTIR confirmed the presence of saturated hydrocarbons, hydroxyl, methylene, and carbonyl functional groups in both extracts.

### 3.2. Characterization of Emulsions

For clarity and consistency, the formulations discussed herein will be referred to as follows: Cream A corresponds to the formulation containing 70% freeze-dried *A. glutinosa* extract, Cream B contains 35% freeze-dried *Alnus glutinosa* extract, Cream C comprises 30% freeze-dried Alnus extract, and Cream D refers to the formulation incorporating the freeze-dried ethanolic extract of *Alnus glutinosa.* This standardized naming will be used throughout the text to facilitate discussion.

#### 3.2.1. Stability of pH and Viscosity

**Figure 4:**
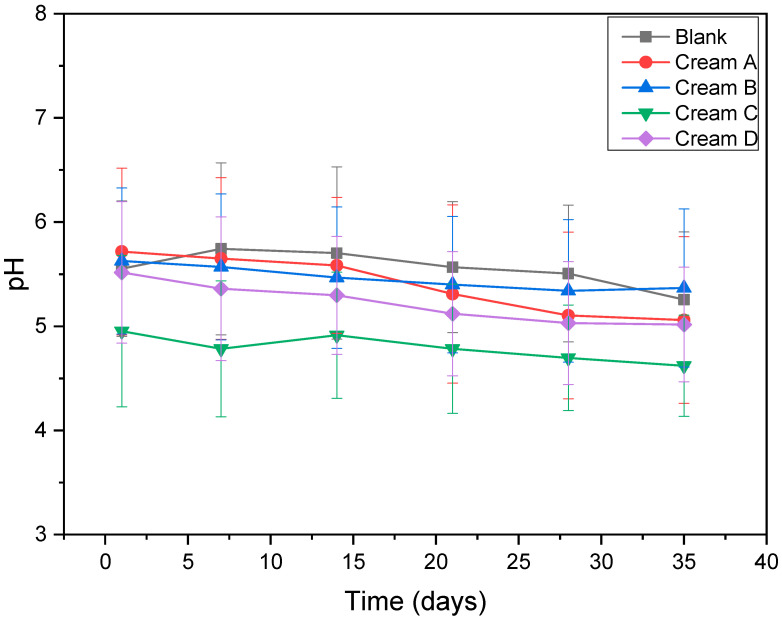
pH measurements of the prepared creams over 35 days of storage.

The pH values of the prepared creams were monitored over a 35-day period to assess stability (Figure 4). Data were analyzed using repeated measures ANOVA to evaluate the effect of time within each formulation (*p* = 0.047). Post hoc pairwise comparisons with Tukey’s correction revealed no significant changes in pH at days 7 and 14 compared to day 1 (*p* > 0.05). However, significant decreases in pH were observed from day 21 onwards (*p* < 0.001). Despite these statistical differences, the actual pH reductions were minor, indicating only slight acidification during storage. The stability in pH is likely due to the mildly acidic nature of key ingredients such as stearic acid, beeswax, shea butter, and xanthan gum. In contrast, formulations containing *Alnus glutinosa* extracts showed a continuous, gradual decrease in pH over time, which was significantly different from the blank emulsions at corresponding time points (*p* < 0.05). These results suggest the extracts contribute to progressive acidification over the storage period.

**Figure 5:**
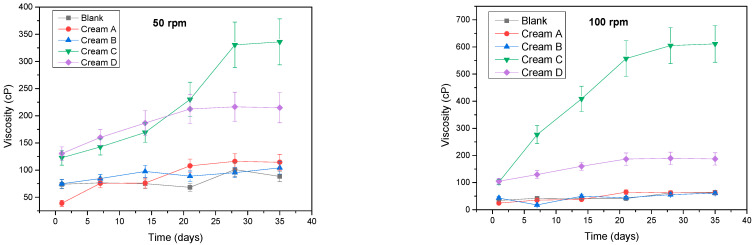
Rheological profiles of the prepared emulsions measured at 50 and 100 rpm over 35 days.

The rheological behavior of the prepared emulsions was evaluated over a 35-day period at shear rates of 50 and 100 rpm (Figure 5) and revealed important insights into the flow behavior of the formulation. The change in viscosity with increasing shear rate suggests non-Newtonian characteristics, typical of many emulsions and complex fluids. Specifically, the observed decrease in viscosity at higher shear rates indicates shear-thinning behavior, where the internal structure of the formulation breaks down under shear, leading to easier flow. This behavior is advantageous for topical creams as it allows easier spreading during application while maintaining stability at rest [22,23].

Both shear rates showed significant effects of time (*p* < 0.001) and cream type (*p* < 0.001). Specifically, the blank emulsion, cream A and cream B did not significantly differ from one another over time, whereas creams C and D did. At 50 rpm, creams C and D already differed significantly from the blank emulsion and from creams A and B, with these differences becoming larger over time. Though cream C did have a significant higher viscosity of 330 and 335 cP than cream D, measured at 216 and 215 cP at day 28 (*p* < 0.001) and day 35 (*p* < 0.001), respectively, no overall difference was observed between the two formulations across the entire study period (*p* = 0.172). At 100 rpm, however, creams C and D differed significantly when examined over the entire timespan (*p* < 0.001). The blank emulsion displayed consistently low viscosity throughout the study, with only a slight increase by day 28, reflecting a lack of structural complexity. Cream A showed a steady increase in viscosity from day 1 to day 21, followed by a plateau, suggesting network development within the emulsion matrix. Cream B, with a lower aqueous extract concentration, presented irregular viscosity values, particularly at higher shear rates, indicating weak structural formation. Cream C recorded the highest viscosity among all formulations, rising steadily until day 21 before stabilizing. Cream D followed a similar trend to Cream C but with slightly lower viscosity values. Both Cream C and D demonstrated consistent shear-thinning behavior, confirming pseudoplastic flow characteristics.

#### 3.2.2. Determination of CIELAB Values

**Figure 6:**
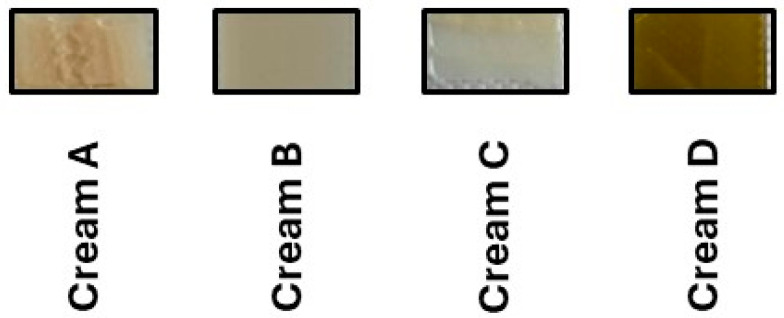
Color observation of the prepared creams.

The colorimetric analyses of the creams revealed a significant overall effect on CIELAB values and color strength (L*: *p* < 0.001; a*: *p* < 0.001; b*: *p* < 0.001; C: *p* < 0.001, Table 5 and Figure 6). More specifically, Cream A, containing approximately twice the extract amount (70%), exhibited the highest L* value (*p* < 0.001), reflecting increased lightness, whereas a* values were significantly higher in Creams B and C compared to the rest of the formulations, respectively. This suggests that extract concentrations influence the cream’s color by increasing lightness at high levels and redness at moderate levels. Cream D, formulated with ethanolic extract, displayed the highest a* value of 6.08 ± 0.17 (*p* < 0.001) as well as the highest C value, i.e., 18.80 ± 0.74 (*p* < 0.001), producing a darker yellow-brown hue. These results indicate a pronounced shift along the red–green axis, with greater redness and color saturation, resulting in a darker yellow-brown hue, consistent with the presence of colored compounds extracted by ethanol. The elevated a* and C values suggest that the ethanolic extract contributes to stronger pigmentation, likely due to bioactive or chromophoric constituents affecting the cream’s appearance [24,25]. In contrast, Creams B and C, formulated with aqueous extracts at varying concentrations, did not statistically differ from each other in terms of their a* (*p* = 0.509) and C values (*p* = 0.920), resulting in pale beige to light yellow hues.

#### 3.2.3. Antioxidant Properties

**Figure 7:**
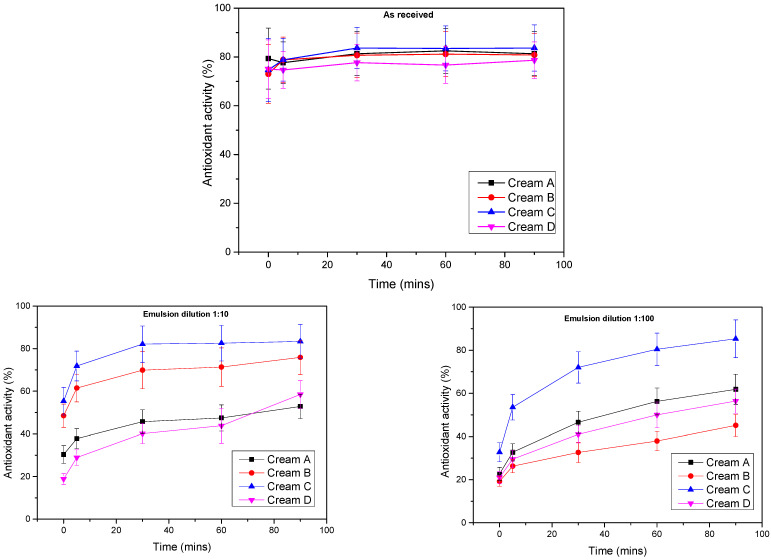
Antioxidant activity of the prepared creams measured over 90 min under three conditions: undiluted (as received), 1:10 dilution, and 1:100 dilution.

Antioxidant activity of the creams was assessed over a 90-min period under three conditions: undiluted (as received), 1:10 diluted, and 1:100 diluted (Figure 7).

In the undiluted state, all extract-rich creams exhibited high initial DPPH scavenging activity, attributable to their elevated phenolic content. At t = 0, inhibition values ranged from approximately 73% to 79%. A significant effect of time was observed (*p* < 0.001), though no group effect over time was detected. Antioxidant activity increased in all creams, with significant differences compared to t = 0 emerging at 30 min (*p* < 0.018) and beyond, indicating relatively rapid reaction kinetics.

For the 1:10 dilution, initial inhibition values ranged from 19% to 55%. Both a significant time effect (*p* < 0.001) and a group effect over time (*p* < 0.001) were observed. The time effect was consistent across all groups, while the group effect reflected creams B and C having significantly higher antioxidant activity than creams A and D (*p* < 0.001). However, no significant difference was observed between creams A and B (*p* = 0.332).

For the 1:100 dilution, initial inhibition values ranged from 19% to 33%. As with the 1:10 dilution, both a significant time effect (*p* < 0.001) and group effect over time (*p* < 0.001) were observed. While all groups showed increasing antioxidant activity over time, the group effect was attributable solely to cream C, which exhibited significantly higher activity than all other formulations (*p* < 0.001).

## 4. Discussion

The extraction and purification of phytochemicals from plant materials are influenced by several factors, including extraction temperature, time, solvent concentration, and polarity. Due to the diverse chemical nature of phytochemicals, their solubility varies across solvents with different polarities. Consequently, no single solvent can effectively extract the full spectrum of phytochemical and antioxidant compounds present in plants [26]. According to various studies, the extractive yield and antioxidant activity of phenolic compounds in plant material are significantly affected by solvent polarity. The greater yield of ethanol extractives compared to water extractives observed in this study is consistent with the solvent’s intermediate polarity, which enables more efficient extraction of a wider range of compounds. Extraction efficiency depends on the match between the solvent polarity and the polarity of the target compounds, rather than solely on absolute solvent polarity. Ghasemzadeh et al. [27] reported that water-ethanol (50:50 *v*/*v*) mixtures yielded higher total phenolics and flavonoids than absolute ethanol, regardless of extraction method. Similarly, Ghaffar and Perveen [28] found that methanol–water mixtures improved extraction yields, phenolic content, and free radical scavenging activity. These findings highlight the advantage of combining polar and less polar solvents to improve extraction efficiency. Overall, the results reinforce the importance of selecting appropriate solvents and techniques to maximize phytochemical recovery.

The visible spectral range (400–700 nm) is informative for identifying chromophoric compounds such as phenolics, flavonoids, and anthocyanins, which are prevalent in plant extracts. The stronger absorbance values in Soxhlet extracts suggest a more effective recovery of these compounds, consistent with its known exhaustive extraction capabilities. The peak near 665 nm in most extracts likely corresponds to chlorophyll A, indicating its co-extraction during the process. Additional peaks around 470 nm and 536 nm are associated with carotenoids and anthocyanins, respectively [29]. The reduced absorbance observed in microwave-assisted extraction may result from shorter exposure times or thermal degradation of sensitive compounds, highlighting the importance of optimizing conditions to preserve chromophores.

The phytochemical profile obtained through LC–HRMS reflects the complex and bioactive-rich nature of the *A. glutinosa* Soxhlet extracts. The identification of compounds such as gallic acid, quinic acid, and neochlorogenic acid highlights the high antioxidant potential of the extracts. These particular phenolic acids possess polar functional groups—multiple hydroxyl and carboxyl moieties—which enhance their solubility in water and explain their abundance in the aqueous extract [30]. The absence of most diarylheptanoids, previously reported in *A. glutinosa* bark, is likely due to the exclusive use of leaf material in this study, where these compounds may be naturally absent or fall below the detection threshold [31]. The presence of both major bioactive constituents and trace-level secondary metabolites demonstrates the value of detailed extraction and analytical techniques in mapping the full therapeutic potential of plant-derived ingredients.

The FTIR analysis of *Alnus glutinosa* extracts in both water and ethanol enabled the identification of saturated hydrocarbons, methylene groups, hydroxyl and carbonyl functional groups. The differences observed in FTIR spectra between water and ethanol extracts reflect the influence of solvent polarity on hydrogen bonding and spectral resolution. Water, a highly polar protic solvent, facilitates extensive and diverse hydrogen bonding, leading to broader and less defined O–H stretching bands in the 3700–3000 cm^−1^ region. Ethanol, with a lower dielectric constant and reduced hydrogen-bonding capacity, produced sharper O–H bands at slightly higher wavenumbers, indicating a more uniform bonding environment [32]. Broadening of absorption bands and reduced peak resolution in aqueous extracts are also likely due to the poor solubility of certain compounds, such as anthranoids, in water. As extract concentration increases, hydroxyl bands become more intense and shift toward lower wavenumbers, consistent with increased hydrogen bonding. Conversely, ethanol enhances spectral clarity and peak separation due to its moderate polarity and better solubilizing capacity for a wider range of phytochemicals [33].

Maintaining pH stability in cosmetic emulsions is essential for product safety, efficacy, and skin compatibility, with the ideal topical range being 4.5 to 6.0 [19]. The slight acidification observed in extract-containing formulations is consistent with the chemical nature of plant-derived phenolic compounds, which can oxidatively degrade to release weak organic acids [34]. Such behavior is common in natural formulations rich in bioactive compounds like flavonoids, tannins, and diarylheptanoids, which are prone to hydrolytic and oxidative transformations over time [35]. The pH shift toward acidity aligns with literature reports on botanical emulsions [10,36], underscoring the dynamic interactions between extract components and emulsion matrices during storage. While these changes are expected in natural systems, monitoring them is crucial to ensure product performance and consumer safety. The statistical analysis revealed a significant effect of time on pH, as indicated by the Greenhouse–Geisser corrected *p*-value of 0.047, while no significant variations (*p* > 0.05) were observed in the tested samples.

Viscosity stability is critical for the usability, shelf life, and performance of cosmetic emulsions, particularly in ensuring spreadability and consumer satisfaction [37]. Blank emulsion and Creams A and B did not yield any significant variations in 50 and 100 rpm (*p* = 1). The observed shear-thinning response across formulations, especially in Creams C and D which significantly varied (*p* ≤ 0.01) from the rest of the emulsions, is indicative of desirable pseudoplastic behavior common in well-structured, plant-based emulsions [38]. The variations in viscosity are closely linked to extract concentration and the solvent system used, where higher phenolic content likely contributes to stronger hydrogen-bonding networks and enhanced matrix integrity. Creams enriched with *A. glutinosa* extracts, particularly Cream C, maintained rheological consistency and demonstrated optimal structural performance over time. These findings align with previous research on botanical emulsions [39], supporting the potential of *A. glutinosa* extracts in creating stable, consumer-friendly cosmeceutical formulations.

The deeper coloration of Cream D is attributed to the superior solubility and extraction capability of ethanol for chromophoric polyphenols such as flavonoids, tannins, and diarylheptanoids [35]. These compounds possess conjugated systems that not only enhance pigmentation but also contribute significantly to antioxidant and photoprotective activity. Similar findings have been reported by Wathoni et al. [40] who observed a direct correlation between polyphenol content and visual intensity in plant-based emulsions. Furthermore, the consistent color trends observed—particularly the heightened pigment intensity in Cream C—highlight the dual role of color as both an esthetic and functional indicator. Darker emulsions are typically richer in bioactive compounds and thus offer improved therapeutic potential, aligning with the overall goals of cosmeceutical formulation.

Free radicals are among the major skin aging factors, and antioxidants are therefore common functional cosmetic ingredients [41,42]. Extracts of *A. glutinosa* contain phenolics that exhibit excellent free-radical scavenging activity. *A. glutinosa* bark, indeed, possesses high total phenolic content (up to ~71% *w*/*w* as gallic acid equivalents) and exhibits high antioxidant activity [14]. The statistical analysis revealed a strong time effect in the antioxidant activity (%) in all cases as indicated by the Greenhouse–Geisser value (*p* < 0.01). The strong initial DPPH inhibition observed in the undiluted samples confirms the high antioxidant capacity of *A. glutinosa* extract-enriched creams, in line with reported data on polyphenol-rich plant extracts [43,44,45]. The rapid radical quenching seen within the first few minutes suggests a high concentration of reactive antioxidant moieties, such as phenolic hydroxyl groups, capable of donating hydrogen atoms or electrons to stabilize DPPH radicals [9,44]. At 1:10 dilution, the marked differences in antioxidant performance between high-extract and low-extract formulations became more pronounced. Creams with higher extract concentrations (A and D) maintained substantial scavenging capacity, whereas Creams B and C showed significantly limited (*p* < 0.05) radical neutralization. This suggests that extract concentration directly influences antioxidant efficiency, likely due to insufficient phenolic content in lower extract formulations to drive the DPPH reaction to completion. Possible interference from the cream matrix (e.g., emulsifiers, oils) at this dilution may also hinder reactivity [45]. At 1:100 dilution, antioxidant efficacy declined, as expected when the antioxidant dose falls below the threshold needed to fully quench the DPPH radicals [46]. The dual-phase kinetic pattern—rapid initial inhibition followed by a slower increase—matches prior models of phenolic radical scavenging observed by Angeli and coworkers [47]. These results also align with findings from Mishra et al. [48] who reported that antioxidant effectiveness is concentration-dependent and influenced by the matrix environment. Overall, these findings reinforce the role of *A. glutinosa* extracts as potent antioxidant agents in topical formulations. Their activity provides supportive evidence for potential anti-aging and skin-protective effects.

The scientific concern regarding artificial sunscreen chemicals and their side effects (skin irritation, hormonal disruption, and environmental toxicity) has driven science towards natural sunscreens [49,50,51,52,53]. Phenolic compounds found in *A. glutinosa* have been shown to act not only as UV absorbers but also as radical scavengers that mitigate ultraviolet (UV)-induced oxidative stress, enhancing the photostability and efficacy of sunscreen formulations [35,41,53]. In vitro cell assays play a crucial role in photoprotection research by providing controlled, reproducible systems to evaluate the cellular response to UV radiation and the efficacy of photoprotective agents. These assays allow for detailed assessment of cell viability, DNA damage, oxidative stress, and apoptosis in skin-related cells such as keratinocytes and fibroblasts. Future research should include detailed investigations of skin permeation and bioavailability of the active compounds from *Alnus glutinosa* emulsions using clinical studies, in vitro or ex vivo skin models, or Strat-M^®^ membranes to better understand their absorption and efficacy in topical applications. Additionally, comparative studies with other well-known natural antioxidants and UV-protective extracts would provide valuable insights into the relative effectiveness of *A. glutinosa*, helping to position it more clearly within the natural cosmeceutical market.

## 5. Conclusions

In this preliminary study, *Alnus glutinosa* extracts were, to the best of our knowledge, incorporated into cosmetic emulsions for the first time, with their composition, physical stability and antioxidant capacity systematically evaluated. Through the comparison of various extraction methods, solvents, and their integration into cosmetic formulations, the research identified the most effective conditions for maximizing benefits and ensuring formulation stability.

A variety of extraction procedures were implemented, and among them, Soxhlet extraction using ethanol—owing to its relatively lower polarity compared to water—proved to be the most effective solvent, with the highest extraction rate of phytochemicals (42.5%). LC-HRMS Orbitrap analysis confirmed the identification and quantification of several bioactive compounds of interest, which have been reported to possess strong antioxidant and photoprotective properties. As expected, emulsions with higher concentration of extracts exhibited greater antioxidant activity, with DPPH scavenging activity over 85% for diluted samples, which also remained at high levels even for higher dilutions. Stability studies ensured that all formulations were in the approved pH range (between 5 and 6) after the 35-day-testing period and exhibited good rheological properties, such as pseudoplasticity and long-term viscosity. Finally, colorimetric analysis supported the efficacy of the extracts, showing that emulsions with more intense coloration—reflecting higher phenolic content—were associated with increased antioxidant activity.

The findings of this preliminary work suggest that the rich phytochemical content of *Alnus glutinosa* extracts supports their potential as natural, multifunctional ingredients for the preparation of potential antioxidant and sun-protective cosmeceutical formulations. Overall, this study provides an preliminary assessment of alnus enriched cosmeceutical formulations through physicochemical characterization. Although natural extracts, such as *A. glutinosa*, are generally regarded as safe for dermal applications, additional experiments—such as biocompatibility testing with human skin cell lines, three-dimensional skin models, and cellular assays for ROS scavenging and UV photoprotection—would further substantiate the current preliminary findings. Incorporating these experiments in future work will reinforce the safety and efficacy evidence, ultimately providing a more comprehensive and scientifically robust evaluation of the studied formulations.

## Figures and Tables

**Table 1:** Experimental Conditions for Soxhlet Extraction.

Process	Temperature (°C)	Time (h)	Solvent
Soxhlet 1	70	3	Water
Soxhlet 2	90	3	Water
Soxhlet 3	70	1	Ethanol

**Table 2:** Summary of the substances utilized for the emulsions’ fabrication.

Sample Name	Blank	Emulsion A	Emulsion B	Emulsion C	Emulsion D
**Ingredients (%)**	**Water phase (75%)**				
Water	70	0	35	40	35
Glycerin	3.5	3.5	3.5	3.5	3.5
Xanthan gum	1.5	1.5	1.5	1.5	1.5
EDTA	0.5	0.5	0.5	0.5	0.5
Soxhlet 1	0	70	35	30	0
Soxhlet 3	0	0	0	0	35
	**Oil Phase (23%)**				
Olive oil	13	13	13	13	13
Cetyl alcohol	2	2	2	2	2
Cetearyl alcohol	2	2	2	2	2
Polysorbate 60	2	2	2	2	2
Shea butter	2	2	2	2	2
Steatic acid	2	2	2	2	2
Beeswax	2	2	2	2	2
	**Additives (2%)**				
Phenoxyethanol	1	1	1	1	1
Ethylhexylglycerin	1	1	1	1	1

**Table 3:** Extractive yield (%) of different extraction methods.

Extraction Method	Extractive Yield (%)
Ultrasound_25 °C	8.20 ± 0.35
Ultrasound_70 °C	14.60 ± 0.50
Soxhlet 1	18.40 ± 0.70
Soxhlet 2	24.90 ± 0.60
Soxhlet 3	42.50 ± 1.10
Microwave	4.80 ± 0.20

**Table 4:** Qualitative and quantitative analysis of *A. glutinosa* Sohxlet extracts by LC—Orbitrap-HRMS analysis.

Compound	Formula	Calc. MW	*m*/*z*	RT [min]
(1E)-1,7-bis(4-hydroxyphenyl)hept-1-en-3-one	C_19_H_20_O_3_	296.14198	295.13471	7.996
12-oxo Phytodienoic Acid	C_18_H_28_O_3_	292.20499	291.19752	1.331
15-Deoxy-Δ12,14-prostaglandin J2-2-glycerol ester	C_23_H_34_O_5_	390.23984	391.24712	1.836
3-(4-{[1,3-Dihydroxy-1-(4-hydroxy-3-methoxyphenyl)-2-propanyl]oxy}-3-methoxyphenyl)propyl 6-deoxy-alpha-L-mannopyranoside	C_26_H_36_O_11_	524.22731	523.22004	5.701
3,4,5-trihydroxycyclohex-1-ene-1-carboxylic acid	C_7_H_10_O_5_	174.0525	173.04517	0.843
3-Methoxy-5,7,3′,4′-tetrahydroxy-flavone	C_16_H_12_O_7_	316.0593	317.06685	7.304
Adenosine	C_10_H_13_N_5_O_4_	267.0977	268.10498	0.814
Asiatic acid	C_30_H_48_O_5_	488.35155	487.34427	12.551
Azelaic acid	C_9_H_16_O_4_	188.10472	187.09744	6.111
Cafestol	C_20_H_28_O_3_	316.20256	317.20984	11.684
Caffeic acid	C_9_H_8_O_4_	180.04196	179.03468	4.541
Citric acid	C_6_H_8_O_7_	192.02685	191.01957	0.825
Corchorifatty acid F	C_18_H_32_O_5_	328.22593	327.21865	7.641
D-(-)-Fructose	C_6_H_12_O_6_	180.0631	179.05575	0.769
D-(-)-Quinic acid	C_7_H_12_O_6_	192.06315	191.05587	0.819
D(+)-Phenyllactic acid	C_9_H_10_O_3_	166.0626	165.05533	4.999
Docosahexaenoic acid ethyl ester	C_24_H_36_O_2_	356.27268	357.27996	12.17
Gallic acid	C_7_H_6_O_5_	170.02114	169.01386	0.816
Genistein	C_15_H_10_O_5_	270.05373	269.04629	7.428
Gentisic acid	C_7_H_6_O_4_	154.02613	153.01878	0.858
L-Phenylalanine	C_9_H_11_NO_2_	165.07974	166.08702	0857
L-Tyrosine	C_9_H_11_NO_3_	181.07474	182.08203	0.862
Luteolin	C_15_H_10_O_6_	286.0486	285.04132	7.271
Miquelianin	C_21_H_18_O_13_	478.07619	477.06891	5.675
N-[4-cyano-1-(4-fluorophenyl)-1H-pyrazol-5-yl]cyclohexanecarboxamide	C_17_H_17_FN_4_O	312.13715	311.12988	6.301
Naringenin	C_15_H_12_O_5_	272.06939	271.0621	6.599
Neochlorogenic acid	C_16_H_18_O_9_	354.09627	353.08899	1.889
Oleanolic acid	C_30_H_48_O_3_	456.36174	455.35446	13.408
Pinolenic acid	C_18_ H_30_O_2_	278.22554	301.21483	12.729
Quercetin-3β-D-glucoside	C_21_H_20_O_12_	464.09733	463.08966	5.794
α-Linolenic acid	C_18_H_30_O_2_	278.22546	279.23273	11.736
(1E)-1,7-bis(4-hydroxyphenyl)hept-1-en-3-one	C_19_H_20_O_3_	296.14198	295.13471	7.996
12-oxo Phytodienoic Acid	C_18_H_28_O_3_	292.20499	291.19752	11.331
15-Deoxy-Δ12,14-prostaglandin J2-2-glycerol ester	C_23_H_34_O_5_	390.23984	391.24712	10.836
3-(4-{[1,3-Dihydroxy-1-(4-hydroxy-3-methoxyphenyl)-2-propanyl]oxy}-3-methoxyphenyl)propyl 6-deoxy-alpha-L-mannopyranoside	C_26_H_36_O_11_	524.22731	523.22004	5.701

**Table 5:** Measurements obtained from the spectrophotometric color analysis.

Sample	L* (Lightness)	a* (Red–Green)	b* (Yellow-Blue)	C (Chroma)	h° (Hue Angle)	R% (Reflectance)	K/S (Color Strength)
**Cream A**	56.04 ± 0.23	−4.14 ± 0.44	14.40 ± 0.30	14.88 ± 0.22	108.12°	7.44 (400 nm)	5.76
**Cream B**	48.03 ± 1.46	−1.46 ± 1.68	11.91 ± 0.33	11.98 ± 0.32	96.13°	10.84 (482 nm)	2.05
**Cream C**	48.64 ± 1.28	−0.28 ± 0.99	9.68 ± 1.11	11.72 ± 0.58	93.45°	5.73 (409 nm)	1.29
**Cream D**	48.63 ± 0.56	6.08 ± 0.17	17.67 ± 0.83	18.80 ± 0.74	71.46°	4.27 (410 nm)	10.73

## Data Availability

The original contributions presented in this study are included in the article/Supplementary Materials. Further inquiries can be directed to the corresponding authors.

## Supplementary Materials

The following supporting information can be downloaded at: https://www.mdpi.com/article/10.3390/antiox14111275/s1. Figure S1: Compound Discoverer workflow of HPLC/MS analysis. Table S1: Plant extracts_LC_MS.

## Abbreviations

The following abbreviations are used in this manuscript:

*A. glutinosa*	*Alnus glutinosa*
DPPH	(2,2-diphenyl-1-picryl-hydrazyl-hydrate)
EtOH	Ethanol
BS	Blank sample
