# Supplementary figures and images for: From Plant to Skin: Exploring Alnus glutinosa Extracts for Cosmeceutical Applications
Source: Antioxidants (Basel). 2025 Oct 23;14(11):1275. doi: 10.3390/antiox14111275 (PMC12649570; doi:10.3390/antiox14111275)

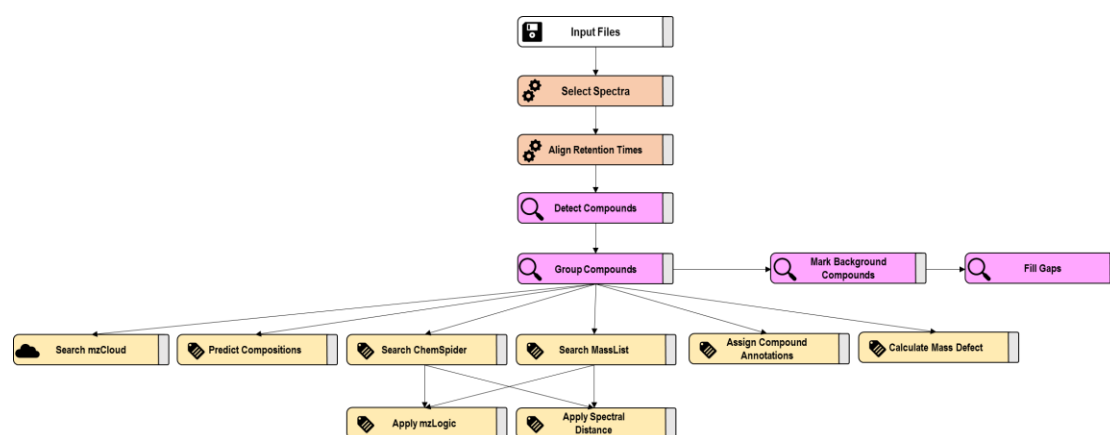

Figure S1: Compound Discoverer workflow of HPLC/MS analysis.

Supplement: Supplementary file 1 [file antioxidants-14-01275-s001.zip › Supplementary file.pdf]
